# Cu_2_O nanoparticles synthesis by microplasma

**DOI:** 10.1038/srep07339

**Published:** 2014-12-05

**Authors:** ChangMing Du, MuDan Xiao

**Affiliations:** 1 Guangdong Provincial Key Laboratory of Environmental Pollution Control and Remediation Technology, School of Environmental Science and Engineering, Sun Yat-Sen University, Guangzhou 510275, China

## Abstract

A simple microplasma method was used to synthesize cuprous oxide (Cu_2_O) nanoparticles in NaCl–NaOH–NaNO_3_ electrolytic system. Microplasma was successfully used as the cathode and copper plate was used as the anode. The Cu_2_O products are characterized by X–ray powder diffraction (XRD), field emission scanning electron microscope (FESEM) and transmission electron microscope (TEM). The results show that the morphology of Cu_2_O nanocrystals obtained by this technology is mainly dependent on the electrolytic media, stirring, current density and reaction temperature. The uniform and monodisperse sphere Cu_2_O nanoparticles with the size about 400 ~ 600 nm can be easily obtained in H_2_O–ethylene glycol mix–solvent (volume ratio 1:1) and appropriate current density with stirring at room temperature. In addition, the possible mechanism has been reported in the article. And the average energy consumed in producing 1 g Cu_2_O nanoparticles is 180 kJ. For the flexibility and effectiveness of this microplasma technology, it will have broad application prospects in the realm of nanoscience, energy and environment.

In this work, a simple microplasma electrochemical technique is reported, where the microplasma takes place at the gas–solution interface as the cathode for the synthesis of cuprous oxide nanoparticles. Therefore, much interest has been focused on exploring the effects of various process parameters such as the type of electrolyte, stirring, pH value, current density and temperature on the growth of Cu_2_O nanostructures. Moreover, the possible formation mechanism for the cuprous oxide nanoparticles synthesized by the newest plasma–liquid electrochemical technology is discussed.

In recent years particularly, nanoparticles have received considerable attention for its fascinating properties and potential applications. As an important p–type semiconductor with a band gap of 2.17 eV^1^,^2^, cuprous oxide (Cu_2_O) is a promising material which has potential applications in electrode materials^3^, solar energy conversion^4^,^5^, sensors^6^ and catalysts^7^,^8^,^9^,^10^. In particular, it has potential applications in photon catalytic degradation of organic pollutants under visible light^11^. Furthermore, major interesting characteristics of Cu_2_O are inexpensive, low toxicity, readily available and good environmental acceptability^12^,^13^,^14^.

Up to now, Cu_2_O nanostructures with various shapes have been synthesized by different methods including hydrothermal method^15^,^16^,^17^,^18^, electrochemical rout^11^,^19^,^20^, chemical vapor deposition of precursors^21^, solution synthesis method^22^ and sonochemical method^23^. However, so far, no report has been researched on the synthesis of Cu_2_O by plasma–liquid electrochemistry technology, needless to say a low energy and microscale microplasma. Microplasma is a special subdivision of electrical discharge formed in electrode geometries where at least one dimension is less than 1 mm^24^. Microplasmas have attracted enormous interest from the plasma organization due to their characteristics of small physical size, excimer generation^25^,^26^,^27^, atmospheric pressure stability^28^ and non–equilibrium thermodynamics^29^,^30^. These properties make microplasmas suitable for a wide range of applications, including medicine, gas treatment, textiles, surface modification and nanofabrication^31^.

## Experimental Section

### Experimental Apparatus and Materials

**Figure 1:**
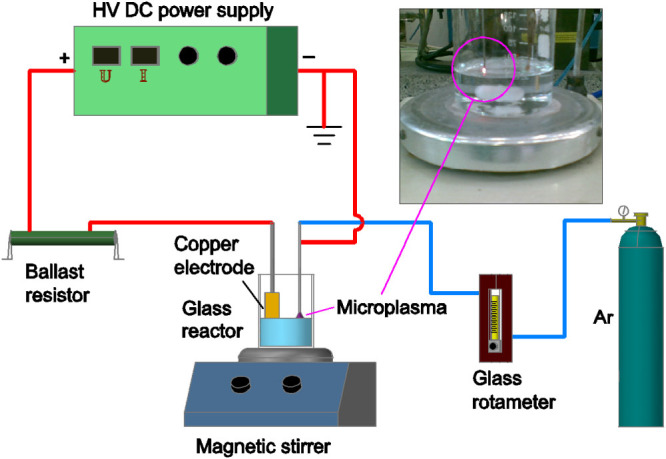
The experimental apparatus diagram of microplasma electrochemical synthesis of Cu_2_O nanoparticles.

The experimental apparatus for microplasma electrochemical synthesis of Cu_2_O nanoparticles is shown in Figure 1. A stainless steel tube (0.7 mm inside diameter, 8 cm length) was positioned 3 cm away from the copper electrode (1 cm width, 3 cm length, immersion area is 1 cm^2^) with a gap of 2 mm between the tube end and the liquid surface. Argon gas flow was coupled to the tube and controlled by a glass rotameter at 60 ml/min. The stainless steel tube acted as the cathode and the copper sheet as the anode. The reactor was made of common glass, with inner diameter of 5.5 cm and length of 8.5 cm. The copper anode was polished and washed with distilled water, and then immerged into electrolyte containing 150 g/L NaCl, 1 g/L NaOH and 1.3 g/L NaNO_3_ with the distilled water or H_2_O–ethylene glycol as the solvent. All chemicals were commercially available in analytical and guaranteed grade. When a high voltage (~2000 V) was applied, the microplasma formed at the gas–solution interface and then kept stable by a ballast resistor (R = 50 kΩ) and lowering the voltage to a certain current. During the preparation, the solution was gently stirred with a magnetic stirrer for acquiring the best production. The microplasma–assisted electrolysis was performed at different process conditions for 20 min. At the end of the synthesis, the sediments were centrifuged and washed with deionized water and ethanol for several times. Subsequently, the obtained products were dried in a vacuum oven at 60°C for 6 h.

### Analysis

The crystalline phase of obtained Cu_2_O nanoparticles was examined by an X–ray diffractometer (D/Max–IIIA, Ragiku). Their morphology, particle size and microstructure were characterized by field emission scanning eletron microscope (FESEM, JEOL JSM-6330F) and transmission electron microscope (TEM, FEI Tecnai G2 Spirit, 120 kV).

## Results and Discussion

### Effects of electrolyte on the morphology of Cu_2_O nanoparticles

**Figure 2:**
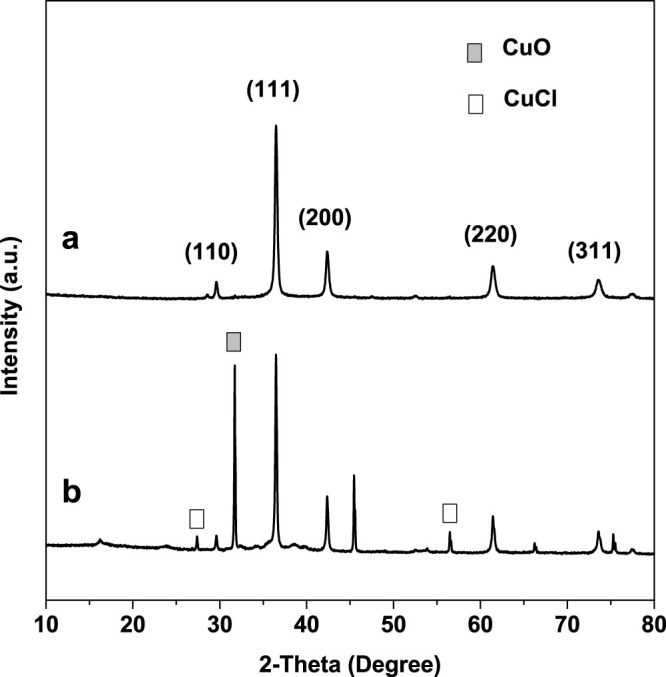
The XRD patterns of the products prepared by microplasma electrochemical method in different electrolyte for 20 min: (a) H_2_O–ethylene glycol (volume ratio 1:1) and (b) pure distilled water. (14 mA/cm^2^ of current density, at room temperature, without stirring).

**Figure 3:**
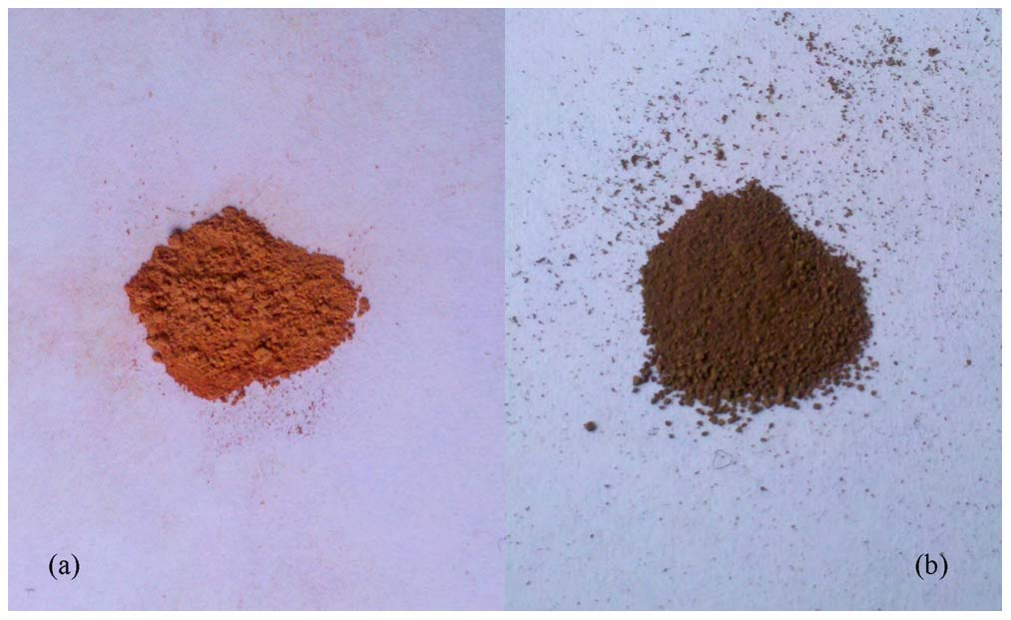
The pictures of products prepared by microplasma in different electrolyte: (a) H_2_O– ethylene glycol (1:1) and (b) pure distilled water.

The effects of electrolyte on the results of Cu_2_O nanoparticles prepared by microplasma electrochemical method were firstly investigated. The XRD patterns of the Cu_2_O synthesized in different electrolytic media (H_2_O and H_2_O–ethylene glycol (volume ratio 1:1, the volume fraction of ethylene glycol is 50%)) under the same conditions in the 2θ range of 10–80° were shown in Figure 2. It is clearly show that Figure 2(a) contains five peaks that are in well agreement with those for Cu_2_O nanocrystals obtained from the International Center of Diffraction Data card (ICDD, formerly JCPDS No. 05–0667). However, according to Figure 2(b), there were a lot peaks appeared which indicated that the products prepared in a pure water solvent also contained CuO and CuCl. This difference could visually be seen from the color of products that prepared in various electrolytes. As shown in Figure 3, the color of pure nanoparticles synthesized in a mix electrolyte were orange, however, the color of impure one which prepared in pure water was darker.

**Figure 4:**
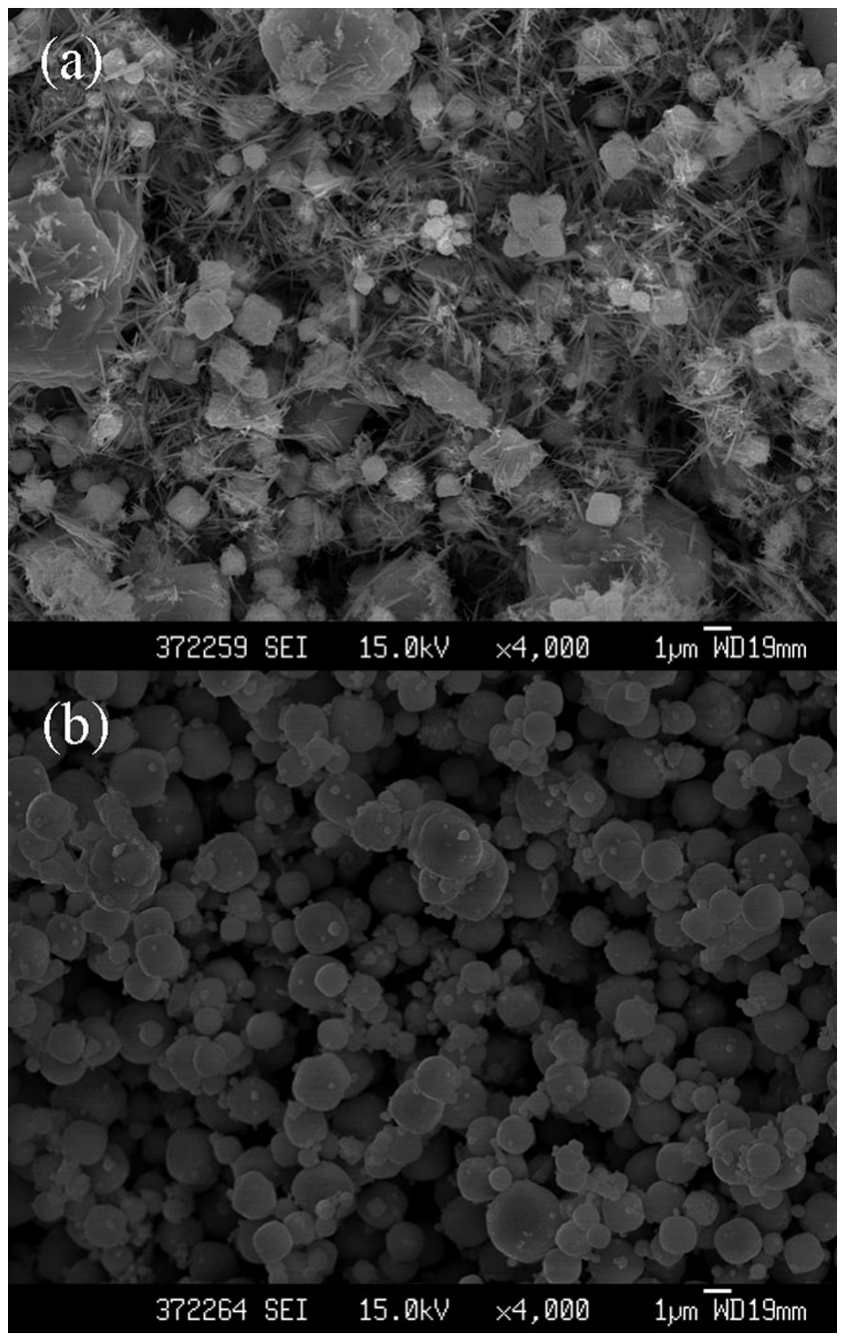
The FESEM images of the Cu_2_O samples prepared in different electrolytic media for 20 min: (a) H_2_O; (b) H_2_O–ethylene glycol (volume ratio 1:1). (14 mA/cm^2^ of current density, at room temperature, without stirring).

Figure 4 shows the SEM images of the produced nanoparticles with different solvents (H_2_O and H_2_O–ethylene glycol (1:1)) as the electrolytic media. It could be observed that varying electrolyte result in different morphology of the obtained products. As can be seen in Figure 4(a), lots of irregular shape structures were synthesized when pure distilled water acted as the solvent. It could be further indicated that other materials may generated in this case. By comparison, in H_2_O–ethylene glycol (1:1) mix solvent, the Cu_2_O crystals exhibit spherical structure with the diameter size ranging from 0.2 to 2 μm (Figure 4(b)).

The XRD and SEM results mentioned above obviously manifest that the effects of electrolyte on the morphology of Cu_2_O is enormous. The H_2_O–ethylene glycol electrolytic media not only can keep off the generation of undesired materials such as CuO and CuCl but also can effectually control the form of Cu_2_O crystals.

### Effects of stirring on the morphology of Cu_2_O nanoparticles

**Figure 5:**
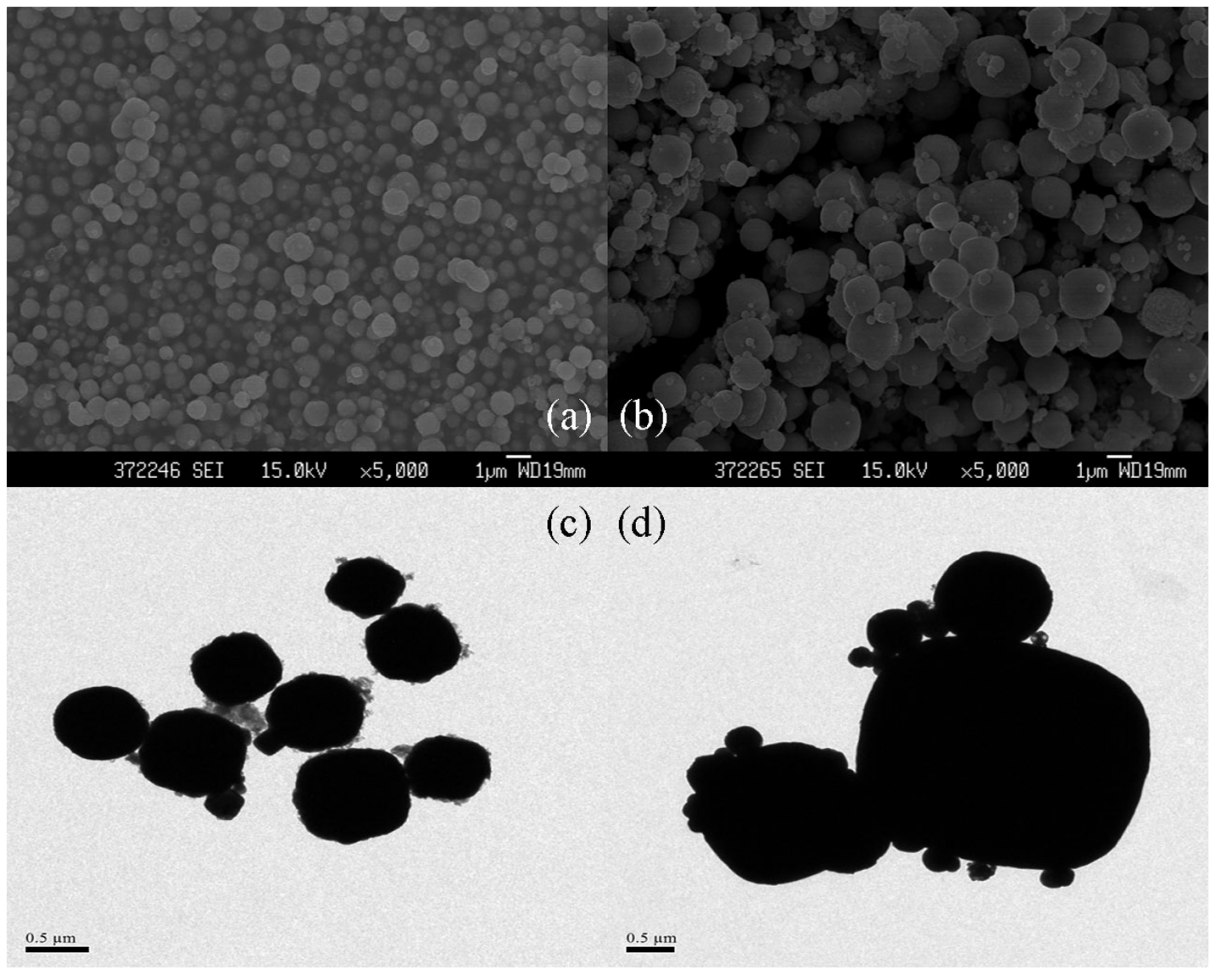
The FESEM and TEM images of the Cu_2_O nanoparticles prepared with stirring ((a) and (c), respectively) and without stirring ((b) and (d), respectively). (14 mA/cm^2^ of current density, at room temperature).

With H_2_O–ethylene glycol as the electrolyte, the next work is to research the effects of stirring on the morphology of Cu_2_O prepared by microplasma technology. It can be found that using stirrer will not lead to the instability of microdischarge. Then the stirrer was used to produce cuprous oxide under the same conditions (14 mA/cm^2^ of current density, room temperature).The products prepared with stirring or without stirring were characterized by SEM ((a) and (b)) and TEM ((c) and (d)) respectively. The results are shown in Figure 5. The shape of the Cu_2_O nanoparticles in two different situations was both in sphere. As can be seen in Figure 5(a) and 5(c), all the nanoparticles are almost uniformly scattered. However, in Figure 5(b), particles with different size tend to aggregate into foot-like products, which can be clearly seen in Figure 5(d). Moreover, the size of those aggregates is large to 2 μm. By comparison, the sizes of the Cu_2_O sphere nanoparticles are around 600 ~ 800 nm in diameter. This result demonstrates that stirring can greatly make the Cu_2_O nanoparticles grow uniformly and dispersedly and have no influence of microdischarge.

### Effects of current density on the morphology of Cu_2_O nanoparticles

**Figure 6:**
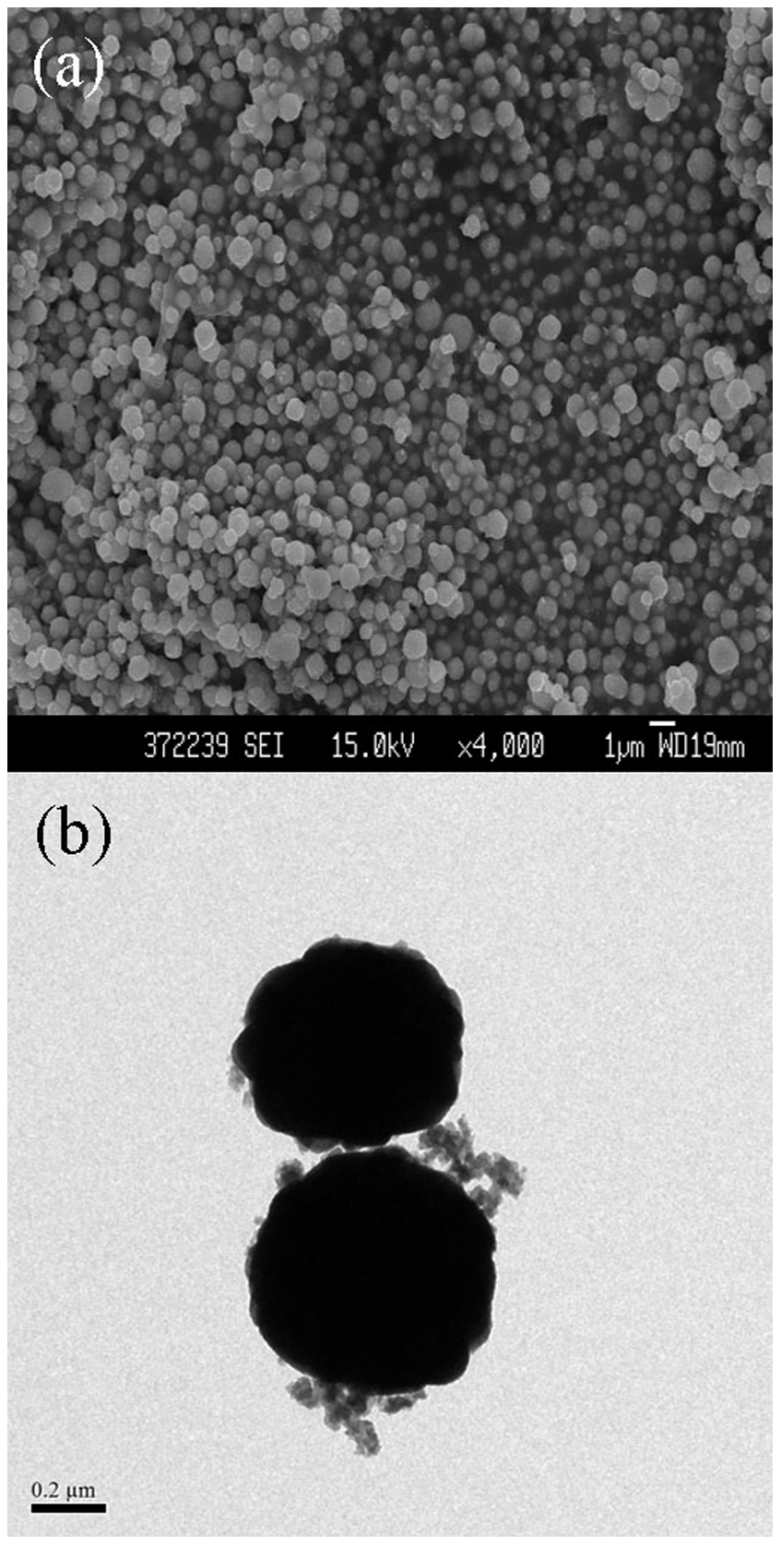
The FESEM (a) and TEM (b) images of the Cu_2_O nanoparticles prepared at current density 10 mA/cm^2^. (at room temperature, with stirring).

In addition, the influence of current density on the growth of cuprous oxide nanoparticles by microplasma electrochemical method was also researched. Firstly, 7 mA/cm^2^ was chosen to run the reaction, while no sediments appeared after micro–discharge for 20 min. Then 10, 14 and 20 mA/cm^2^ was the next consideration value. Finally, it can be found that 20 mA/cm^2^ of current density was so big that the generated high energy would burn out the discharge tube. Therefore, other two values will bring out the results of current density effect on the morphology of Cu_2_O. The FESEM and TEM images of the Cu_2_O nanoparticles synthesized at current density 10 mA/cm^2^ were shown in Figure 6. In comparison, the average diameter of Cu_2_O nanoparticles prepared at 10 mA/cm^2^ was about 400 ~ 600 nm and it is smaller than the above mentioned case at 14 mA/cm^2^ (Figure 5(a) and 5(c)). Moreover, Cu_2_O nanoparticles prepared at 10 mA/cm^2^ was more uniform and regular. This mainly because the formation rate of Cu_2_O nanoparticles is dependent on the current density. When the current density was higher, a very large amount of particles could generate in a short time and get together soon. Hence, it's essential to choose a suitable current density.

### Effects of reaction temperature on the morphology of Cu_2_O nanoparticles

**Figure 7:**
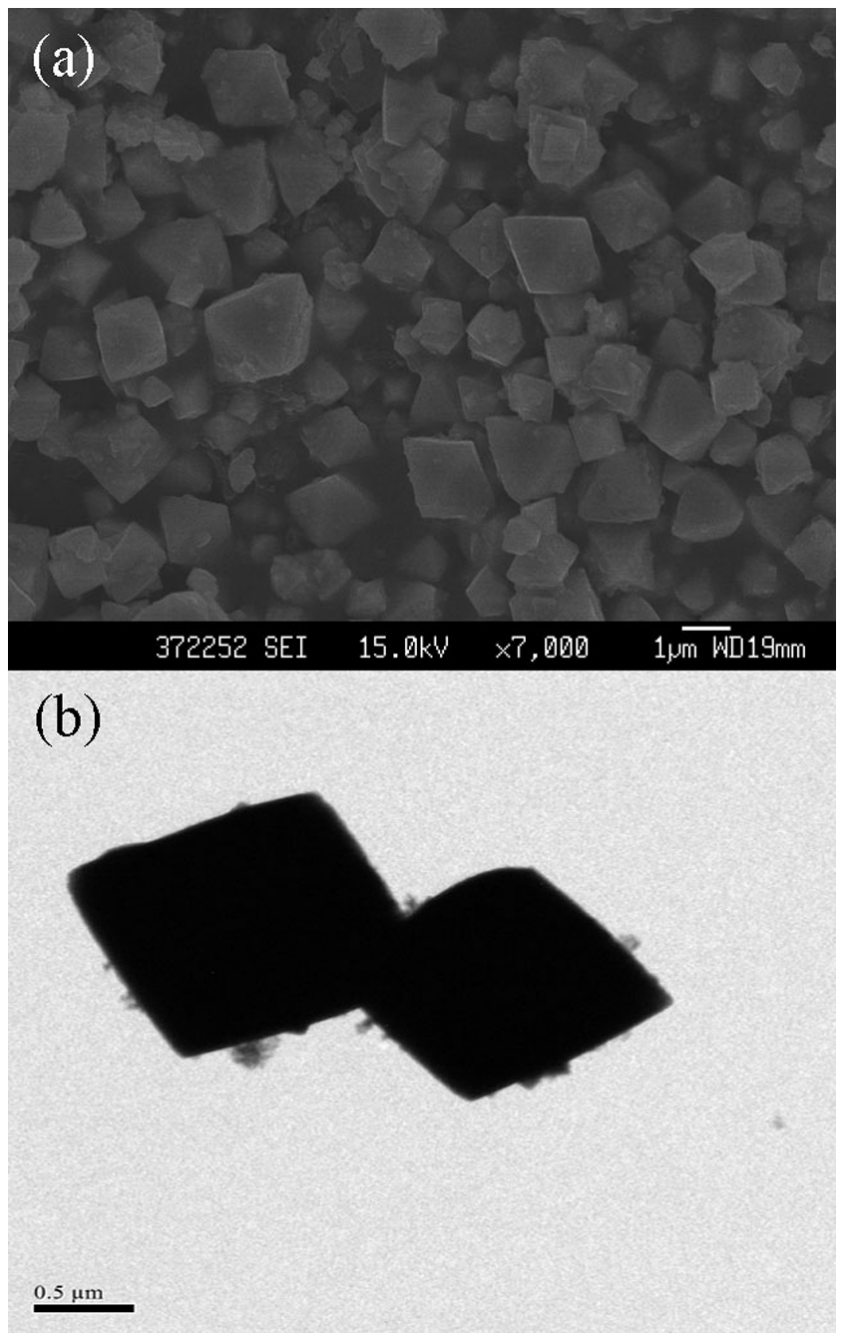
The FESEM (a) and TEM (b) images of the Cu_2_O nanoparticles prepared at 80°C (10 mA/cm^2^ of current density, with stirring).

As a significant thermodynamic parameter, reaction temperature exhibits a considerable influence on the morphology of nanoparticles. A representative FESEM and TEM micrographs of the cuprous oxide nanocrystals prepared at 80°C for 20 min is shown in Figure 7. It is already known that Cu_2_O synthesized at room temperature has a sphere shape, as shown in Figure 5(a) and 5(b). However, with the rising of reaction temperature, morphology of products changed gradually from sphere to octahedron under the same conditions, and the size of nanocrystals was added to 1 μm (Figure 7). Therefore, temperature can not only affect the morphology of Cu_2_O nanoparticles but also change the dimension of them.

**Figure 8:**
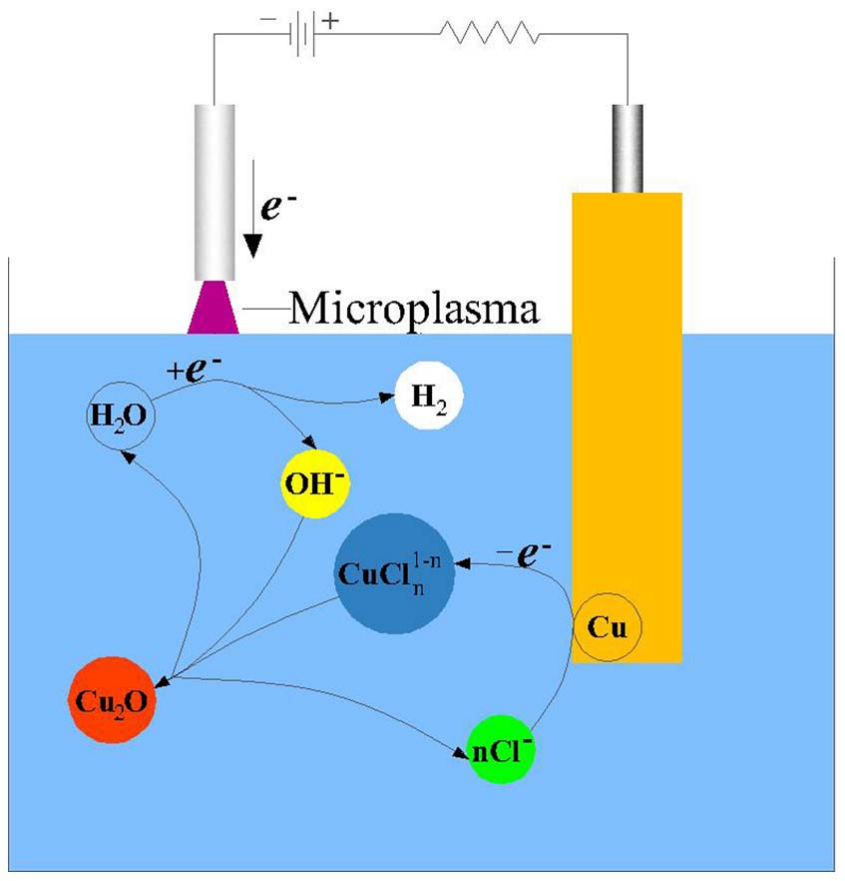
A schematic of microplasma electrochemical synthesis of Cu_2_O nanoparticles.

For the above results, the possible microplasma formation mechanism of Cu_2_O nanoparticles was proposed below. Above all, the measured pH value of the reaction liquid was 12.5 which indicated that this preparation by microplasma is available in strong alkali. Microplasma being the cathode can produce lots of electron to initiate the redox reaction in solution and the microplasma electrochemical reactions are visually depicted in Figure 8 and described as follows^32^: cathodic reaction:


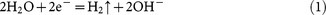
anode reaction:


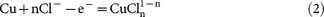
cell reaction:


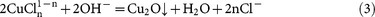
Hence, the total reaction equation is summarized as follow:


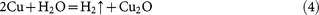
Then the formation of Cu_2_O nanoparticles might undergo nucleation, selective adsorption and growth process. Broadly speaking, the morphology of nanoparticles is mainly determined by its inherent structure and process parameters. As is known, the growth rates vary along the different crystallographic directions. The final morphology will be determined by the fast growth plane^33^. In this research, nanocrystals prepared in all conditions except at 80°C, the presence of ethylene glycol lets the growth rates of all directions of Cu_2_O nanoparticles keep nearly the same, which ultimately result in the generation of solid nanosphere structures. Moreover, the mix electrolytic media makes the dispersion of nanoparticles more effective. In addition, the use of stirring actually impedes the combination between different nanoparticles and prevents the crystal from further growth, and so do the condition of low current density which is mainly because current density can determine the generation rate of Cu_2_O nanocrystals. However, when the reaction temperature rises to 80°C, the surface energy of (111) plane of Cu_2_O crystals reduces and further result in the growth rate along that crystal orientations being lower. As a consequence, the octahedral shape of Cu_2_O nanoparticles is generated with the assistance of microplasma. More importantly, cuprous oxide nanoparticles of different morphology are the vital raw materials for the widespread application domain. It is necessary to know that the average energy consumed in producing 1 g Cu_2_O nanoparticles is 180 kJ. As the flexibility and effectiveness of this microplasma technology, this method can be used in large area such as synthesis of very useful nanomaterials, removal of pollutants, production of hydrogen and so on.

## Conclusion

On the basis of the results of present research it can be concluded that we are able to synthesize different possible Cu_2_O nanoparticles (nanosphere and nanooctahedron) by a very newest and inexpensive method of microplasma electrochemical technology. The microplasma operated at the liquid–gas interface replaces the traditional solid electrode that makes the preparation of cuprous oxide nanoparticles more effective. In particular, our experiment is the first attempt to apply the microplasma into the preparation of Cu_2_O nanoparticles. However, the theoretical fundamental of this method is not well built. Therefore, more efforts should be undertaken to demonstrate the present results to exploit this microplasma technology. More importantly, the prepared Cu_2_O nanoparticles have a widely application prospects in environmental governance such as adsorption of organic pollutants.

## Author Contributions

D.C.M. and X.M.D. wrote the main manuscript text and prepared figures 1–8 together. All authors reviewed the manuscript.
